# Muscle Shortening and Spastic Cocontraction in Gastrocnemius Medialis and Peroneus Longus in Very Young Hemiparetic Children

**DOI:** 10.1155/2018/2328601

**Published:** 2018-05-21

**Authors:** M. Vinti, N. Bayle, A. Merlo, G. Authier, S. Pesenti, J.-L. Jouve, B. Chabrol, J.-M. Gracies, C. Boulay

**Affiliations:** ^1^EA 7377 BIOTN, Université Paris Est Créteil (UPEC), Créteil, France; ^2^AP-HP, Service de Rééducation Neurolocomotrice, Hôpitaux Universitaires Henri Mondor, Créteil, France; ^3^Motion Analysis Laboratory (LAM), Department of Rehabilitation, Azienda Unità Sanitaria Locale Reggio Emilia, S. Sebastiano Hospital, Correggio, Italy; ^4^Gait Laboratory, Pediatric Orthopaedic Surgery Department, Timone Children's Hospital, Marseille, France; ^5^Aix-Marseille University, CNRS, ISM UMR 7287, Marseille, France; ^6^Pediatric Neurology Department, Timone Children's Hospital, Marseille, France

## Abstract

**Objectives:**

Muscle shortening and spastic cocontraction in ankle plantar flexors may alter gait since early childhood in cerebral palsy (CP). We evaluated gastrosoleus complex (GSC) length, and gastrocnemius medialis (GM) and peroneus longus (PL) activity during swing phase, in very young hemiparetic children with equinovalgus.

**Methods:**

This was an observational, retrospective, and monocentric outpatient study in a pediatric hospital. Ten very young hemiparetic children (age 3 ± 1 yrs) were enrolled. These CP children were assessed for muscle extensibility (Tardieu scale *X*_V1_) in GSC (angle of arrest during slow-speed passive ankle dorsiflexion with the knee extended) and monitored for GM and PL electromyography (EMG) during the swing phase of gait. The swing phase was divided into three periods (T1, T2, and T3), in which we measured a cocontraction index (CCI), ratio of the Root Mean Square EMG (RMS-EMG) from each muscle during that period to the peak 500 ms RMS-EMG obtained from voluntary plantar flexion during standing on tiptoes (from several 5-second series, the highest RMS value was computed over 500 ms around the peak).

**Results:**

On the paretic side: (i) the mean *X*_V1-GSC_ was 100° (8°) (median (SD)) versus 106° (3°) on the nonparetic side (*p* = 0.032, Mann–Whitney); (ii) *X*_V1-GSC_ diminished with age between ages of 2 and 5 (Spearman, *ρ* = 0.019); (iii) CCI_GM_ and CCI_PL_ during swing phase were higher than on the nonparetic side (CCI_GM_, 0.32 (0.20) versus 0.15 (0.09), *p* < 0.01; CCI_PL_, 0.52 (0.30) versus 0.24 (0.17), *p* < 0.01), with an early difference significant for PL from T1 (*p* = 0.03).

**Conclusions:**

In very young hemiparetic children, the paretic GSC may rapidly shorten in the first years of life. GM and PL cocontraction during swing phase are excessive, which contributes to dynamic equinovalgus. Muscle extensibility (*X*_V1_) may have to be monitored and preserved in the first years of life in children with CP. Additional measurements of cocontraction may further help target treatments with botulinum toxin, especially in peroneus longus.

## 1. Introduction

In spastic paresis, placing a muscle under tension may increase its cocontraction (antagonistic recruitment during opposite voluntary effort) and the cocontraction of homonymous muscles [1]. Such tension may arise when stretch is applied on a contractured muscle. There have been suggestions in infant paresis that passive muscle extensibility diminishes with age [2], that this gradual loss of extensibility is actually the clinical representation of a true histological muscle disease with myofascial thickening [3], and that this muscle disease may represent a more prominent problem than spasticity [2–6].

Overall, we aimed to contribute to improving treatment selections for plantar flexor shortening and overactivity in very young hemiparetic children. Specifically, our study aimed to investigate the mechanisms underlying limited dorsiflexion during swing phase and equinovalgus [7] at initial contact in children with a spastic hemiparesis. To this end we used noninvasive methods which may be applied in clinical practice.

Using the conditions of the clinical examination at rest, the first goal of the present investigation was to quantify passive muscle extensibility and spasticity in gastrosoleus complex (GSC), to discriminate spasticity and changes in mechanical properties of soft tissue structures [4]. Using the dynamic conditions of gait analysis, the second goal was to measure spastic cocontraction in gastrocnemius medialis (GM) and peroneus longus (PL) during the swing phase of gait, comparing nonparetic and paretic legs using dynamic electromyography (EMG). We subdivided the swing phase into initial, middle, and late thirds to quantify cocontraction across the swing phase progression.

Our hypotheses were that (i) levels of cocontraction in both GM and PL would be higher on the paretic than on the nonparetic side; (ii) muscle contracture would be present by age 5. Regarding GM and PL cocontraction, we expected to observe these two muscles behave differently across the different periods of the swing phase [7], with antagonist activation of PL throughout the swing phase, while that of GM might start only late in swing due to the knee reextension—and therefore to the increasingly stretched position of GM—that occurs at that stage.

This approach could give better insight into the mechanisms of the reduced range of motion in children with a spastic paresis. Clinical implications in rehabilitation might be that (i) muscle extensibility in triceps surae may have to be monitored and preserved in the first five years of life in children with CP by stretching, orthosis, casting, or physical therapy programs, (ii) additional measurements of cocontraction may further help to target treatments with botulinum toxin, especially in PL.

## 2. Methods

### 2.1. Subjects and Clinical Features

**Figure 1:**
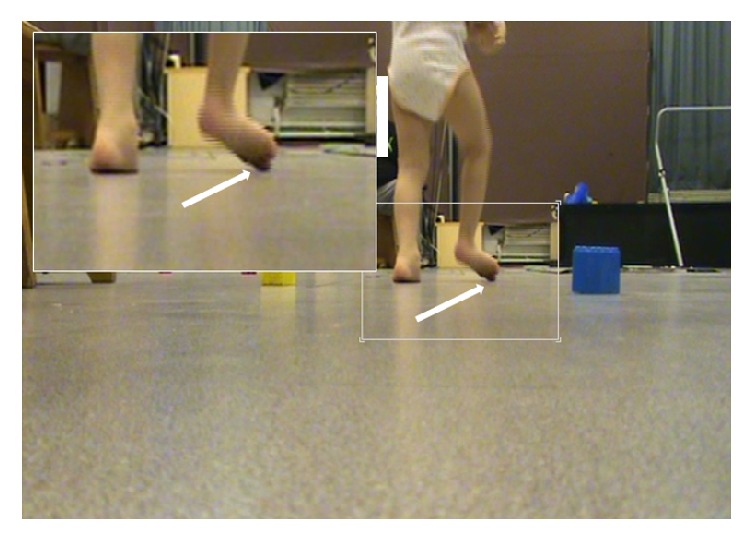
*Posterior-anterior view of the initial contact in a right hemiparetic children*. Criteria for inclusion: diagnosis of right equinovalgus was based on the initial contact by the hallux (white arrow) and/or the head of the first metatarsal.

Ten children (4 girls, 3 (1) years, median (SD); age range 2–5) with cerebral palsy (CP) and spastic hemiparesis (5 left side) type 2 as classified by Winters Jr. et al. (1987) [8] were selected for this study after parental consent (Table 1). Criteria for inclusion were diagnosis of CP made by a pediatric neurologist, age under 6, hemiparetic involvement and equinovalgus deformity clinically confirmed by two investigators, and Gross Motor Function Classification System (GMFCS) 1 or 2. The main criterion for equinovalgus was an initial contact with the hallux or the head of the first metatarsal which allowed determining gait cycle onsets (Figure 1). Criteria for exclusion were a severe fixed equinus deformity due to major triceps surae contracture (range of passive dorsiflexion knee flexed < 80°, 0° being defined as the theoretical position of minimal stretch of the tested muscle, which would correspond to “full” plantar flexion) [9] and inter-limb length discrepancy over 1 cm.

### 2.2. Clinical Assessments

The following clinical measures were collected bilaterally.

(i) The first were steps 2 and 3 of the Five-Step Assessment (FSA) of spastic paresis, an expansion of the Tardieu scale that has been previously validated in children [9, 10], for the soleus (knee flexed) and gastrocnemius muscles (knee extended). Step 2 measures *X*_V1_, which is the fibula-calcaneum angle (angle between the fibula and the posterior half of the external border of the foot) at which further slow-speed passive ankle dorsiflexion would cause pain to the patient or jeopardize joint integrity based on the clinician assessment. *X*_V1_ is taken here as a clinical estimate of the passive extensibility of the gastrosoleus complex, which is constituted by one single joint muscle, the soleus, and two trans-joint muscles, the gastrocnemius medialis and lateralis. *X*_V1_ of soleus is assessed with the knee flexed; *X*_V1_ of GSC is assessed with the knee extended. This measurement of the angle of end-range resistance was carried out on subjects lying in supine position, with the knee flexed and extended, ensuring that no foot pronation occurred during the maneuver. Step 3 measures spasticity, using three parameters: *X*_V3_, angle of catch (ankle angle at which the assessor feels a brisk reaction of the muscle) or clonus when the muscle is stretched at fast speed; *X*, the spasticity angle, defined as the difference *X*_V1_ − *X*_V3_, which reflects the velocity-dependent stretch reflex only (the larger the spasticity angle, the more spastic the muscle); and *Y*, the grade of spasticity (0: no resistance throughout passive movement; 1: slight resistance throughout passive movement with no clear catch at a precise angle; 2: clear catch at a precise angle, interrupting passive movement, followed by release; 3: fatigable clonus (<10 s when maintaining pressure) occurring at a precise angle, followed by release; 4: unfatigable clonus (>10 s when maintaining pressure) occurring at a precise angle) [9].

(ii) We added an exploratory goniometric assessment of *A*_0_ [11–14], the tension threshold, which is the fibula-calcaneum angle at which concomitant palpation of the Achilles tendon feels the first tension upon very slow passive stretch of the plantar flexors. A passive stretch course (PSC) with the knee extended, that is, the difference *X*_V1_ − *A*_0_, was derived.

Repeatability measurements of clinical data (*X*_V1_, *X*_V3_, and *A*_0_ on the paretic side with the knee extended) were performed using the Schwartz et al. protocol [15]: for one hemiparetic child, each parameter was measured 30 times, that is, by 2 observers (CB and GA of the same gait laboratory), during 3 sessions and 5 times per session. The results yielded an intertherapist deviation (InterTherDev = *σ*^InterTher^ in degree), an intersession deviation (InterSessDev = *σ*^InterSess^ in degree), and an intertrial deviation (InterTrialDev = *σ*^InterTrial^ in degree) and concluded with a global evaluation (*r* = *σ*^InterTher^/*σ*^InterTrial^).

In fact the variations of clinical measurement arise from different sources: the intrinsic and extrinsic variability. The intrinsic variability of the clinical parameter is measured by the intertrial deviations. The extrinsic variability is assessed not only by the intersession deviations, which measure the errors introduced when a single therapist (observer) repeats the clinical evaluation, but also with the intertherapist deviations which measure the errors introduced when multiple therapists (observers) measure the clinical parameter. The reliability of a clinical parameter (*X*_V1_, *X*_V3_, and *A*_0_ on the paretic side with knee extended) is measured by the magnitude of the intertherapist deviations which reflect the overall reliability of the clinical parameter and its ratio to intertrial variability (*r* = *σ*^InterTher^/*σ*^InterTrial^). The intertrial error is free of methodological errors and serves as an appropriate baseline for comparisons [15].

### 2.3. Gait Laboratory Evaluation

Gait evaluation involved video and dynamic EMG as children walked barefoot at self-selected speed. The ankle and rearfoot position at initial contact were defined using different points of view, including posterior views that revealed the position of the rearfoot and the area of initial contact (Figure 1). The main criterion for equinovalgus was an initial contact with the hallux or the head of the first metatarsal, which allowed detecting the onset of gait cycles. To identify reproducible cycles for analysis, we used the EMG Easy Report© software (MerloBioEngineering, Italy).

### 2.4. EMG Acquisition

Before positioning the electrodes, the skin was abraded (using first alcohol then Nuprep®), cleansed with isotonic sodium chloride (0.9%), and dried with gauze at the electrode sites. Two Ag-AgCl surface electrodes, 10 mm in diameter, were placed below the fibula head, parallel to the PL fibers, at 25% of the fibula head-lateral malleolus line [16, 17]. The electrodes for GM and tibialis anterior (TA) were placed on the most prominent bulge of the GM and TA muscle along the circular line perpendicular to the vertical line fibula head-lateral malleolus, at the junction between the 1st and 2nd fourth and with the help of muscle echography [16, 17]. The electrode pairs were placed along the fiber direction. Interelectrode distance (center to center distance between the conductive areas of the two bipolar electrodes) was 10 mm, as recommended by SENIAM for noninvasive surface EMG in young children [16]. An elastic tape was used to maintain the electrodes on the skin so the children could move freely with little risk for local wires to pull the electrodes off. EMG data were collected using a wireless system (Wave wireless EMG, Cometa, Italy), acquired at 2000 Hz, amplified (1000x), filtered (10 Hz high-pass filter, 500 Hz low-pass filter), and rectified [17].

The validation procedure to minimize crosstalk between the GM, PL, and TA electrode locations included the following movements in supine position: ankle plantar flexion (knee extended) for GM, forefoot eversion for PL, or inversion for TA. The validation procedure involved observing timing differences of EMG onset between PL, GM, and TA during voluntary contractions [7]. A systematically synchronized EMG onset is consistent with crosstalk between two muscles.

**Figure 2:**
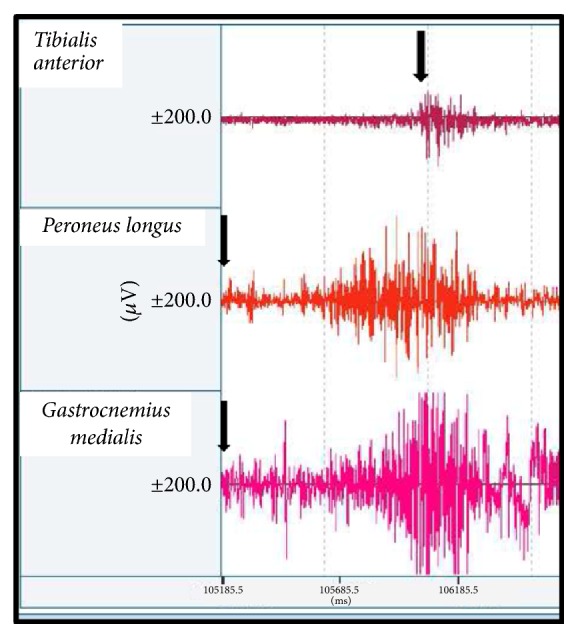
*Example of spastic cocontraction*. The patient, in supine position with knee extended, is asked to produce an ankle dorsiflexion in the paretic side. Spastic cocontraction refers to inappropriate antagonist recruitment (ankle plantar flexors as GM and PL in this example) triggered by the volitional command on an agonist (ankle dorsal flexors as TA). The hypothesis is that a supraspinal factor (misdirected descending drive) is primarily involved and encounters an overall hyperexcitable ankle plantar flexor neuron pool. It takes some times for the patient to initiate dorsiflexor recruitment which is insufficient to counterbalance the ankle plantar flexor movement.

Spastic cocontraction refers to inappropriate antagonist recruitment (in this case the ankle plantar flexors PL and GM) triggered by the volitional command directed to agonist (in this case the ankle dorsiflexor TA) [18]. The evidence for abnormal antagonist cocontraction during active agonist movement in spastic paresis is overwhelming (Figure 2) [18]. Although some cocontraction (simultaneous activity in both agonist and antagonist) is common during normal human movement, in spastic paresis it is present to an excessive degree [18].

To obtain a reference for submaximal plantar flexor EMG, children were asked to stand on tiptoes, using slight manual support on a table: this movement, in closed kinetic chain, best recruited GM and the plantar flexor component of PL. Then bilateral PL and GM EMGs were recorded during gait barefoot at self-selected speed.

### 2.5. EMG Analysis

**Figure 3:**
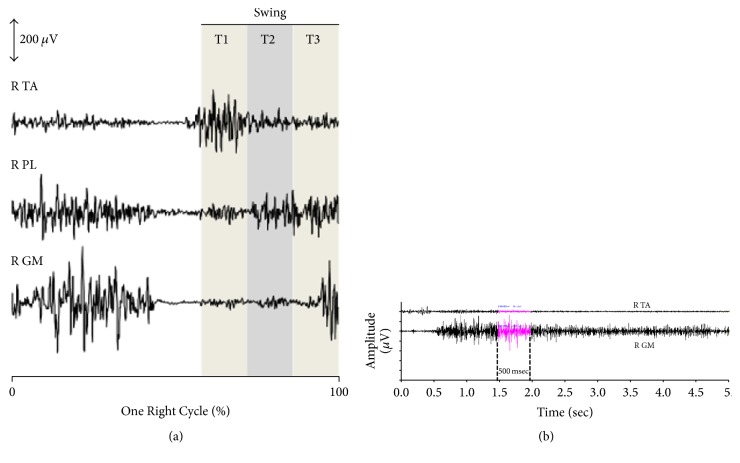
*Individual data from a child with right (R) hemiparesis*. (a) Electromyography (amplitude in *μ*V) of tibialis anterior (R TA), peroneus longus (R PL), and gastrocnemius medialis (R GM) during the swing phase. Subdivision of the swing phase in three equal parts T1, T2, and T3. (b) Calculation of the cocontraction index (CCI). Example of gastrocnemius medialis (R GM) CCI. The reference maximal agonist GM RMS is averaged over the 500 ms interval around the peak voltage during a submaximal voluntary effort selected, that is, standing on tiptoes (5 s): the EMG Easy Report© software (MerloBioEngineering, Italy) detected the peak (144 *μ*V) and calculated the RMS (78 *μ*V) around this peak, that is, between 1.41 s and 1.91 s (500 ms). The antagonist GM RMS is calculated during the swing phase (active ankle dorsi flexor; during swing phase the activation of ankle plantar flexor muscles (GM, PL) means an abnormal premature pattern of activation). The GM cocontraction index (CCI_GM_) is obtained from the ratio RMS GM_antagonist_/RMS GM_agonist_ (during the whole swing phase and each subphase T1, T2, and T3).

For each muscle, the root mean square (RMS) amplitude was computed during the swing phase and each swing subphase, T1, T2, and T3, obtained by dividing the time of swing phase into three equal parts (Figure 3(a)). Numerical values for each stride were computed and exported to a spreadsheet for further analysis. All EMG data management was performed using the EMG Easy Report© software (MerloBioEngineering, Italy). Normalized amplitudes were used to compute the cocontraction index (CCI), as previously described [1]. We calculated a Cocontraction Index (CCI) of each ankle plantar flexor (GM and PL) defined as the ratio of the Root Mean Square (RMS) of a muscle when acting as antagonist to the effort intended (during swing phase, ankle dorsiflexors are active but, depending on its magnitude, the activation of ankle plantar flexor muscles (GM, PL) may represent an abnormal pattern of contraction) to the RMS of the same muscle when acting as an agonist during a submaximal voluntary effort selected, that is, standing on tiptoes (several series of 5 s on tiptoes and the highest RMS value is computed over 500 ms around the peak of EMG) (Figure 3(b)). The cocontraction indices of GM (CCI_GM_) and PL (CCI_PL_) were assessed during the whole swing phase and during each subphase (T1, T2, and T3).

### 2.6. Statistical Analysis

As data distribution failed normality assumption testing (Kolmogorov-Smirnov) in each instance, only nonparametric testing was used throughout this study. Mann–Whitney tests were used to assess differences in the clinical measures of GSC (*X*_V1_, *X*_V3_, *Y*, *A*_0_, and PSC) and in the CCI between the paretic and nonparetic sides. To check for differences in CCI across periods of the swing phase (T1-T2-T3), we first used a Kruskal-Wallis test to look for a side-subphase interaction and then a Wilcoxon test to explore differences between pairs.

Spearman correlations of ranks were used to explore any relationship between the potential predictor age and the dependent variable *X*_V1_ on each side (nonparetic and paretic) and between the three parameters *A*_0_, *X*_V1_, and *X*_V3_. Significance was set at *p* < 0.05. All statistical analyses were conducted with SPSS (18.0) software package.

### 2.7. Ethical Approval

All procedures performed in studies involving human participants were in accordance with the ethical standards of the institutional committee (Aix-Marseille University 2014-11-05-03) and with the 1964 Helsinki declaration and its later amendments.

## 3. Results 

### 3.1. Clinical Results

The clinical characteristics of the children are reported in Table 1, including goniometric assessments of maximal passive ankle dorsiflexion (*X*_V1_) knee flexed and extended, the measures of spasticity angles (*X* = *X*_V1_ − *X*_V3_), and spasticity grades for the GSC (knee extended).

Repeatability measurements were collected for one hemiparetic child: the variability for *X*_V1_ (mean = 8.57°, SD = 1.36°, min = 6°, max = 10°, *σ*^InterTher^ = 0.774°, *σ*^InterSess^ = 0.762°, *σ*^InterTrial^ = 0.628°, and *r* = 1.231), for *X*_V3_ (mean = 3.2°, SD = 1.61°, min = 0°, max = 5°, *σ*^InterTher^ = 0.916°, *σ*^InterSess^ = 0.915°, *σ*^InterTrial^ = 0.863°, and *r* = 1.061), and for A_O_ (mean = 4.57°, SD = 1.14°, min = 3°, max = 6°, *σ*^InterTher^ = 0.647°, *σ*^InterSess^ = 0.624°, *σ*^InterTrial^ = 0.618°, and *r* = 1.048).

### 3.2. EMG Results

#### 3.2.1. Cocontraction Index (CCI)

**Figure 4:**
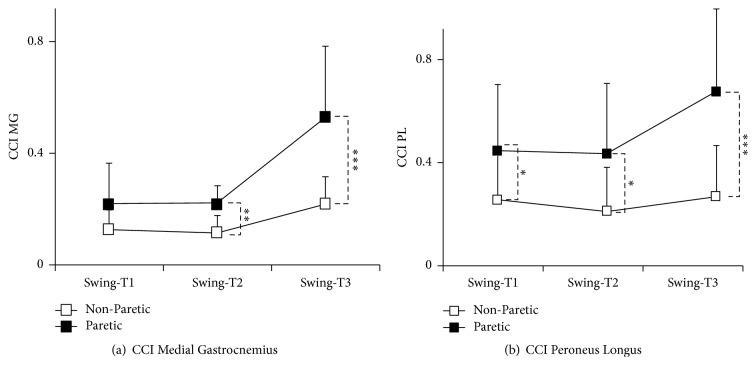
*Cocontraction indices (CCI) during the three parts of the swing phase (T1, T2, and T3) on the paretic and nonparetic sides*. Gastrocnemius medialis (a); Peroneus longus (b). ^*∗*^*p* < 0.05; ^*∗∗*^*p* < 0.01; ^*∗∗∗*^*p* < 0.001.

About 65 ± 40 (mean ± SD) gait cycles per subject were available for analysis (657 gait cycles were studied in total). When considering the whole swing phase, there was a significant difference between the paretic and nonparetic sides for the cocontraction index of both muscles, with CCIs that were about doubled on the paretic side (Wilcoxon: CCI_GM_, 0.30 (0.2) versus 0.15 (0.09), *p* < 0.01; CCI_PL_, 0.52 (0.3) versus 0.24 (0.17), *p* < 0.01) (Figure 4). Statistical analysis for subperiods (T1, T2, and T3) of the swing phase showed an interaction between the side and the period of swing for the cocontraction index in both muscles (Kruskal-Wallis, CCI_GM_, and CCI_PL_, *p* < 0.0001). Post hoc comparisons showed significant differences between paretic and nonparetic sides, with cocontraction increasing on the paretic versus the nonparetic side in the mid- and end periods of swing (CCI_GMT2_, *p* < 0.01; CCI_GMT3_, *p* < 0.001, Wilcoxon) for GM (Figure 4(a)) and in each of the three phases for PL (Figure 4(b)) (CCI_PLT1_, *p* = 0.03; CCI_PLT2_, *p* = 0.014; and CCI_PLT3_, *p* < 0.001).

#### 3.2.2. Passive Muscle Extensibility

**Figure 5:**
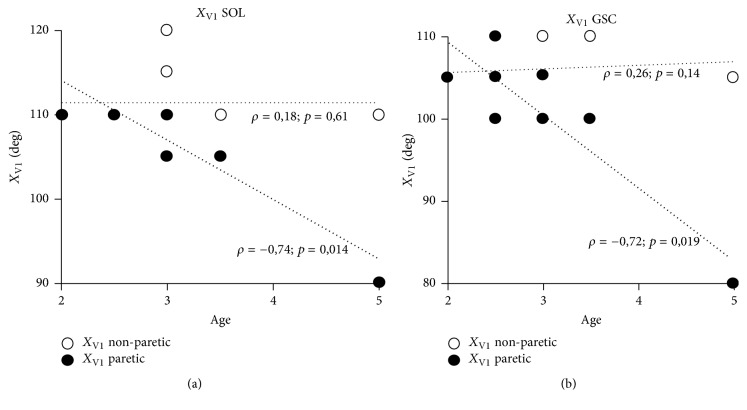
*Scatter plot and Spearman correlation coefficient of ankle dorsal flexion and age*. *X*_V1_, angle of arrest at slow speed, is taken as a clinical estimate of the passive extensibility of the gastrosoleus complex (GSC) which is constituted by one monoarticular muscle, the soleus (SOL), and two biarticular muscles, the gastrocnemius medialis and lateralis. *X*_V1_ of soleus (monoarticular muscle) is assessed in knee flexed; *X*_V1_ of GSC (biarticular muscle) is assessed in knee extended. The linear regressions (Spearman rank correlation) between age and *X*_V1_ of paretic side are statistically significant for *X*_V1_ SOL (*ρ* = −0.74; *p* = 0.014) and *X*_V1_ GSC (*ρ* = −0.72; *p* = 0.019). In the nonparetic side, the correlations are not significant: *X*_V1_ SOL (*ρ* = +0.18; *p* = 0.61) and *X*_V1_ GSC (*ρ* = +0.26; *p* = 0.14). The ankle plantar flexor extensibility of the hemiparetic side decreased with age meaning an early stiffness of GSC. Note: there are no 20 points on each scatter plot because there is only 5 values for *X*_V1_ SOL and 4 values for *X*_V1_ GSC; moreover there are superpositions because some children are the same age (Table 1). For the same age, *X*_V1_ is equal between paretic and nonparetic: 5 children for *X*_V1_ SOL and *X*_V1_ GSC.

Table 1 shows that passive extensibility (*X*_V1_) of soleus (knee flexed) and of GSC (knee extended) were different across sides (*p* < 0.05, Mann–Whitney). There was also a trend for a difference between the two sides for *A*_0_. There was no difference in amplitudes of the passive stretch curve (PSC: 15.0° (8.8) versus 10° (5.0)) between sides. Spearman correlations of ranks suggested associations between age and *X*_V1_ for soleus (*ρ* = −0.74, *p* = 0.014) and GSC (*ρ* = −0.72, *p* = 0.019) (Figure 5), as well as for *X*_V3_ GSC (not illustrated). *X*_V1_ SOL correlated with *X*_V1_ GSC (*ρ* = 0.66; *p* = 0.039). For *Ao* GSC, the paretic and nonparetic sides are correlated (*ρ* = 0.91; *p* = 0.001).

## 4. Discussion

This study in very young hemiparetic children shows an abnormally high level of cocontraction in gastrocnemius medialis and peroneus longus during the swing phase of gait on the paretic side with respect to the nonparetic side. While antagonist action of gastrocnemius medialis was initially low but significantly increased during the middle and the end of the swing phase, excessive antagonist action of peroneus longus started from the initial swing phase: GM and PL; thus both have an abnormal antagonist activity during swing phase. The study also shows marked reduction of gastrosoleus extensibility by age 5 in children with infant hemiparesis.

### 4.1. Limitations of the Study

This study is cross-sectional, with a small study sample comprising only one child older than four. The very young age (3 (1) years, mean (SD)) of our hemiparetic children allows comparing the paretic versus nonparetic side because the paretic feet remain relatively flexible and not fixed as described by Sees and Miller [19].

Moreover, during the maneuver of passive ankle dorsiflexion, any forefoot pronation—which we tried to avoid in the study procedure—can provoke dorsiflexion in the mid-foot and in the ankle. In healthy adults, foot deformity can affect the assessment of the triceps surae muscle-tendon unit length-ankle joint angle during plantar and/or dorsiflexion [20]. But, in our hemiparetic children, the feet arches were not modified by an abnormal raising of the first metatarsus or a stiffness of plantar aponeurosis which minimizes the Iwanuma et al.'s assumption [20] in our study.

One observer (CB) practiced the following clinical procedure: one hand maintains a physiological (5°) hind-foot valgus; the other hand maintains the forefoot without pronation and both hands make dorsiflexion of calcaneum, and another observer (GA) assesses the magnitude of calcaneum dorsiflexion angle on the goniometer according to Gracies et al.'s procedure [9].

We feel that the differences in passive muscle extensibility between the paretic and nonparetic sides corroborate the suggestion of a relation between age and loss of passive muscle extensibility in this population, in line with recent reports [21–23].

The repeatability measurements using Schwartz et al.'s protocol [15] (for the clinical parameters: *X*_V1_, *X*_V3_, and *A*_*O*_) focused on the intertrial variation along with intersession and intertherapist errors. The intertrial variation measures the intrinsic repeatability of clinical parameters, thereby serving as an important reference level to which the extrinsic sources of error can be compared. Intersession and intertherapist errors are extrinsic, arising from various methodological sources including palpation, alignment processes of the goniometer, and the position of hind foot. The repeatability measurements of the clinical parameters (*X*_V1_, *X*_V3_, and *A*_*O*_) are acceptable and show good reliability not only in the magnitude of the intertherapist error (<1°) but also in the ratio to intertrial error (for *X*_V1_: *r* = 1.231; for *X*_V3_: *r* = 1.061; for *A*_*O*_: *r* = 1.048). The ratio of intertherapist to intertrial error (*r*) reveals the influence of experimental (extrinsic) variability: in our study, the small value of *r* shows that reproduction of these clinical parameters was easy [15]. The repeatability procedure used in this study could be improved by increasing the number of hemiparetic children.

Figure 2 illustrates spastic cocontraction. The patient, in supine position with knee extended, is asked to produce an ankle dorsiflexion in the paretic side. Spastic cocontraction refers to inappropriate antagonist recruitment (of the ankle plantar flexors GM and PL in this example) triggered by the volitional command on an agonist (ankle dorsal flexors as TA). The hypothesis is that a supraspinal factor (misdirected descending drive) is primarily involved and encounters an overall hyperexcitable ankle plantar flexor neuron pool. The time it takes for the patient to initiate ankle dorsiflexor (TA) recruitment is insufficient to counterbalance the ankle plantar flexor torque; this is an argument against crosstalk.

The way we determined the submaximal reference EMG of the plantar flexors (during standing on tiptoes) to calculate cocontraction indices may be also subject to criticism since this standing on tiptoes may not involve maximal plantar flexor (GM and PL) recruitment on either side. In addition, children with hemiparesis tend to underload the paretic foot when standing on tiptoes, which would then underestimate the reference submaximal EMG in the calculation of cocontraction indices.

Electrodes with 10 mm diameter may imply considerable spatial averaging and filtering of the EMG [24]. It is necessary to be aware of this filtering which makes any comparison with results obtained using electrodes of different geometry and distance difficult [24].

### 4.2. Methodology of Cocontraction Measurement in Very Young Hemiparetic Children

The quantified findings on cocontraction in the present study confirm those of Boulay et al. [7], which were based only on the timing of EMG onset. Spastic cocontraction has been defined as a misdirected supraspinal command to an antagonist muscle, increased by its stretched position [1, 18, 25]. Adverse GSC overactivity upon active dorsiflexion during the swing phase of gait has been reported elsewhere in infant hemiparesis, with particular focus on its premature onset [26–33]. However, while there have been concerns about PL overactivity [28], EMG explorations of PL activity have been scarce [30]. Quantification of plantar flexor cocontraction remains largely unachieved in clinic or even lab settings, especially in very young hemiparetic children [7]. Issues in quantifying cocontraction in this specific population arise, among others, from the difficulty in scaling cocontracting plantar flexor EMG to reference activation levels recorded during isometric maximum contractions.

EMG amplitude is typically scaled to activation levels recorded during a maximal isometric contraction or a maximal EMG (Mmax) evoked by supramaximal stimulation of the afferent nerve, which may be painful in some subjects [34]. In this study, we used a reference value of plantar flexor activity during a specified state (on tiptoes). However imperfect this strategy may be, a number of arguments support this choice, including the lack of ability to understand tasks of maximal effort in very young children, especially in case of additional cognitive impairment [35]. In addition, isometric tasks performed in supine position are not reliable in very young children, especially with infant paresis [36]. Finally, poor selective muscular control in CP led to preferring a functional test in standing position where much of the segmental and descending pathways activation are likely to produce close to maximal muscular contraction [30]. In this way, measurement of plantar flexor cocontraction proved feasible in very young hemiparetic children although its level of reliability will need confirmatory studies.

### 4.3. Antagonist Cocontraction of Gastrocnemius Medialis and Peroneus Longus during the Three Periods of the Swing Phase

Excessive plantar flexor activation in swing phase has already been described between ages 2 and 7, which has been associated with the overall motor weakness documented in CP and with the higher energy costs during treadmill walking [27, 35–38]. The present quantification of GM antagonist cocontraction supports previous findings on the late latency of GM activation during swing phase, on the paretic side relative to the nonparetic side [7]. Antagonist cocontraction of the GM was significantly increased during the middle and end part of the swing phase, which might be due to the knee reextension that occurs during late swing of gait. Such knee extension places the GM under increased tension by lengthening (eccentric contraction), which could facilitate GM motor neurons and exacerbate the impact of any misdirected descending signal on these motor neurons, akin to the known spastic cocontraction phenomenon described in adult hemiparetic patients [18, 25]. As for the PL, this muscle is normally at rest during the swing phase of gait [7]. In this study, antagonist PL action was increased on the paretic side throughout the whole swing phase. A mostly central origin is likely to explain these findings as in the spastic cocontraction phenomenon demonstrated in adult hemiparetics [1, 18, 25].

### 4.4. Muscle Stiffening in the Very Early Life of CP Children: Which Clinical Parameter Best Evaluates This Phenomenon?

The Tardieu scale parameters *X*_V1_, angle of arrest of the ankle when plantar flexor muscles are stretched at very slow speed, and *X*_V3_, angle of catch (angle at which the assessor feels a brisk reaction of the muscle) or clonus when plantar flexor muscles are stretched at fast speed, have shown reliability in children [9]. Interestingly, the angle at which the examiner felt the first resistance when slowly stretching GSC (*A*_0_) failed to correlate with age or any other clinical parameter tested (*X*_V1_, *X*_V3_) and correlated only with the same parameter on the other side. Our findings suggest that GSC in infant paresis stiffens as early as the first couple of years of life and that the clinical assessment of parameter *X*_V1_ may best detect and evaluate this phenomenon [2]. The reliability and value of the parameter *A*_0_ will need further evaluation.

These potentially early soft tissue plastic rearrangements may only increase with relative disuse and unloading of the paretic limb during growth [2, 4, 18, 21–23, 39–42]. According to Dayanidhi and Lieber [43], in cerebral palsy, longitudinal sarcomere growth and sectional growth of myofibers may be impaired and modifications of extracellular matrix (fibrotic changes and transcriptional profile) may be the underpinnings of contracture development.

**Figure 6:**
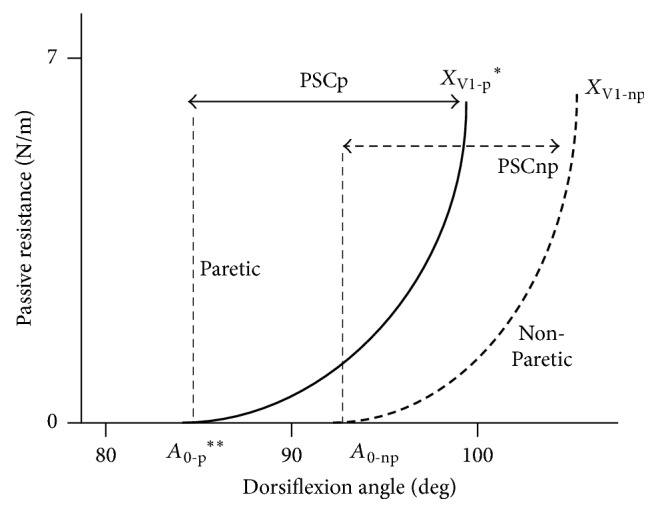
*Proposed model of gastrosoleus complex stiffness*. There is shift towards plantar flexion on the paretic side. The curve is modelled as an exponential relation.* x*-axis (in degrees by goniometry); *y*-axis (in N/m by dynamometry); *A*_0_, tension threshold, that is, dorsiflexion angle at which very slow passive stretch of gastrosoleus complex faces abnormal resistance, detected by the palpation on the Achilles tendon; *X*_V1_, passive extensibility of gastrosoleus complex (V1, slowest stretch velocity possible); PSC, Passive Stretching Course = *X*_V1_ − *A*_0_; p: paretic, np: nonparetic; ^*∗*^*p* < 0.05; ^*∗∗*^*p* < 0.01.

This study also suggested that capacities of GSC extensibility from the tension threshold (*A*_0_) to *X*_V1_ (maximal passive stretch course) were similar between paretic and nonparetic sides. It appears that the PSC is shifted towards plantar flexion on the paretic side [13, 44]; thus contractile efficiency of triceps surae may be shifted towards plantar flexed position (Figure 6) [7, 14, 40–42, 44]. For a highly pennate muscle like the GM, a smaller physiological cross-sectional area implies shortening of the muscle belly [45–49], which could explain the shift of the curve to the plantar flexion, while the slope is not changed or maybe reduced.

## 5. Conclusion

In very young children with hemiparesis and equinovalgus, excessive cocontraction of gastrocnemius medialis and peroneus longus paired with early stiffening of gastrosoleus, to participate in equinovalgus in the swing phase of gait. Understanding the role played by the gastrosoleus and peroneus longus muscles in child equinovalgus might help improve treatment selections for plantar flexor overactivity and shortening in very young hemiparetic children [50].

## Figures and Tables

**Table 1:** Clinical characteristic.

Subject	Gender	Age	Paretic side	Knee flexed		Knee extended								
				Nonparetic	Paretic	Nonparetic	Paretic				Nonparetic		Paretic	
				*X*_V1_	*X*_V1_	*X*_V1_	*X*_V1_	*X*_V3_	*X*_V1_ − *X*_V3_	*Y*	*A*_0_	PSC	*A*_0_	PSC
1	F	2.5	L	110	110	110	110	nd	nd	2	nd	nd	nd	nd
2	M	3	L	115	110	105	100	nd	nd	2	90	15	70	30
3	M	3	R	110	105	105	100	90	10	2	95	10	80	20
4	F	5	L	110	90	105	80	70	10	2	90	15	70	10
5	M	3.5	L	110	105	110	100	80	20	2	95	15	80	20
6	F	3	L	110	110	105	105	85	20	2	100	5	100	5
7	M	2.5	R	110	110	100	100	100	0	0	95	5	95	5
8	M	2	R	110	110	105	105	90	15	2	95	10	95	10
9	M	2.5	R	110	110	105	105	90	15	2	95	10	90	15
10	F	3	R	120	110	110	100	70	30	2	90	20	75	25

*Median*		*3*		*110*	*110*	*105*	*100*	*87.5*	*15*	*2*	*95*	*10*	*80*	*15*
*SD*		*0.8*		*3.4*	*6.3*	*3.2*	*8*	*10.5*	*8.9*	*0.6*	*3.3*	*5*	*11.4*	*8.8*
*np vs p*				*0.032*		*0.032*					*0.08*	*0.39*		

nd: no data; p: paretic; np: nonparetic; *X*_V1_: passive extensibility of Gastrosoleus complex (V1, slowest stretch velocity possible); *X*_V3_: angle of catch of Gastrosoleus complex (V3, fastest stretch velocity possible), *X*_V1_ − *X*_V3_: *X*: spasticity angle; *Y*: grade of spasticity; *A*_0_: tension threshold during a slow passive stretch of the Gastrosoleus complex. PSC: *X*_V1_ − *A*_0_ Passive Stretch Course; age in years; values of *X*_V1_, *X*_V3_, *X*_V1_ − *X*_V3_, *A*_0_, and PSC are expressed in degrees, measured from 0° being the position of minimal stretch of the gastrosoleus complex.

## References

[1] Vinti M., Couillandre A., Hausselle J. (2013). Influence of effort intensity and gastrocnemius stretch on co-contraction and torque production in the healthy and paretic ankle. *Clinical Neurophysiology*.

[2] Alhusaini A. A., Crosbie J., Shepherd R. B., Dean C. M., Scheinberg A. (2010). Mechanical properties of the plantarflexor musculotendinous unit during passive dorsiflexion in children with cerebral palsy compared with typically developing children. *Developmental Medicine & Child Neurology*.

[3] De Bruin M., Smeulders M. J., Kreulen M., Huijing P. A., Jaspers R. T. (2014). Intramuscular connective tissue differences in spastic and control muscle: A mechanical and histological study. *PLoS ONE*.

[4] Gracies J.-M. (2015). Coefficients of impairment in deforming spastic paresis. *Annals of Physical and Rehabilitation Medicine*.

[5] Van Reeth C., Pauwels C., Bayle N., Loche C., Gracies J. (2013). Predominant factors of motor deficiencies in adult spastic paresis: Infant vs adult-acquired lesions. *Annals of Physical and Rehabilitation Medicine*.

[6] Van Reeth C., Pauwels C., Bayle N., Loche C., Gracies J. (2013). Correlation between muscle length, spasticity and motor weakness in adult spastic paresis: Infant vs adult-acquired lesions. *Annals of Physical and Rehabilitation Medicine*.

[7] Boulay C., Pomero V., Viehweger E. (2012). Dynamic equinus with hindfoot valgus in children with hemiplegia. *Gait & Posture*.

[8] Winters T. F., Gage J. R., Hicks R. (1987). Gait patterns in spastic hemiplegia in children and young adults. *The Journal of Bone & Joint Surgery*.

[9] Gracies J.-M., Burke K., Clegg N. J. (2010). Reliability of the Tardieu Scale for Assessing Spasticity in Children With Cerebral Palsy. *Archives of Physical Medicine and Rehabilitation*.

[10] Gracies JM., Bayle N., Vinti M., Alkandari S., Vu P., Loche CM. (2010). Five-step clinical assessment in spastic paresis. *European Journal of Physical and Rehabilitation Medicine*.

[11] Tardieu C., Colbeau-Justin P., Bret M. D., Lespargot A., Huet de la Tour E., Tardieu G. (1976). An apparatus and a method for measuring the relationship of triceps surae torques to tibio-tarsal angles in man. *European Journal of Applied Physiology*.

[12] Tardieu C., Tardieu G., Colbeau-Justin P., Huet de la Tour E., Lespargot A. (1979). Trophic muscle regulation in children with congenital cerebral lesions. *Journal of the Neurological Sciences*.

[13] Tardieu C., Colbeau-Justin P., Bret M. D., Lespargot A., Tardieu G. (1981). Effects on torque angle curve of differences between the recorded tibia-calcaneal angle and the true anatomical angle. *European Journal of Applied Physiology*.

[14] Lespargot A., Renaudin E., Robert M., Khouri N. (1999). Les muscles et les tendons de l'IMOC: examen clinique et données expérimentales. *Motricité Cérébrale*.

[15] Schwartz M. H., Trost J. P., Wervey R. A. (2004). Measurement and management of errors in quantitative gait data. *Gait & Posture*.

[16] Merletti R., Hermens H. (2000). Introduction to the special issue on the SENIAM European Concerted Action. *Journal of Electromyography & Kinesiology*.

[17] Merletti R., Farina D., Gazzoni M., Merlo A., Ossola P., Rainoldi A. (2001). Surface electromyography: A window on the muscle, a glimpse on the central nervous system. *European Journal of Physical and Rehabilitation Medicine*.

[18] Gracies J.-M. (2005). Pathophysiology of spastic paresis. II: emergence of muscle overactivity. *Muscle & Nerve*.

[19] Sees J. P., Miller F. (2013). Overview of foot deformity management in children with cerebral palsy. *Journal of Children's Orthopaedics*.

[20] Iwanuma S., Akagi R., Hashizume S., Kanehisa H., Yanai T., Kawakami Y. (2011). Triceps surae muscle-tendon unit length changes as a function of ankle joint angles and contraction levels: The effect of foot arch deformation. *Journal of Biomechanics*.

[21] Hägglund G., Wagner P. (2011). Spasticity of the gastrosoleus muscle is related to the development of reduced passive dorsiflexion of the ankle in children with cerebral palsy: A registry analysis of 2,796 examinations in 355 children. *Acta Orthopaedica*.

[22] Willerslev-Olsen M., Lorentzen J., Sinkjær T., Nielsen J. B. (2013). Passive muscle properties are altered in children with cerebral palsy before the age of 3 years and are difficult to distinguish clinically from spasticity. *Developmental Medicine & Child Neurology*.

[23] Herskind A., Ritterband-Rosenbaum A., Willerslev-Olsen M. (2016). Muscle growth is reduced in 15-month-old children with cerebral palsy. *Developmental Medicine & Child Neurology*.

[24] Merletti R., Farina D. (2016). *Surface Electromyography: Physiology, Engineering and Applications*.

[25] Vinti M., Bayle N., Hutin E., Burke D., Gracies J.-M. (2015). Stretch-sensitive paresis and effort perception in hemiparesis. *Journal of Neural Transmission*.

[26] Elder GC., Kirk J., Stewart G., Cook K., Weir D., Marshall A. (2003). Contributing factors to muscle weakness in children with cerebral palsy. *Developmental Medicine & Child Neurology*.

[27] Policy J. F., Torburn L., Rinsky L. A., Rose J. (2001). Electromyographic test to differentiate mild diplegic cerebral palsy and idiopathic toe-walking. *Journal of Pediatric Orthopaedics*.

[28] Duchenne (de Boulogne) GB (1872). Impotence fonctionnelle et spasme fonctionnel du long péronier latéral. *Archives Générales de Médecine*.

[29] Hoover G. H., Frost H. M. (1969). Dynamic correction of spastic rocker-bottom foot. Peroneal to anterior tibial tendon transfer and heel-cord lengthening.. *Clinical Orthopaedics and Related Research*.

[30] Fowler E. G., Staudt L. A., Greenberg M. B. (2010). Lower-extremity selective voluntary motor control in patients with spastic cerebral palsy: Increased distal motor impairment. *Developmental Medicine & Child Neurology*.

[31] Crompton J., Imms C., McCoy A. T. (2007). Group-based task-related training for children with cerebral palsy: A pilot study. *Physical & Occupational Therapy in Geriatrics*.

[32] Kuang R. Z., Kalil K. (1990). Specificity of corticospinal axon arbors sprouting into denervated contralateral spinal cord. *Journal of Comparative Neurology*.

[33] Ikeda A. J., Abel M. F., Granata K. P., Damiano D. L. (1998). Quantification of cocontraction in spastic cerebral palsy. *Electroencephalography and Clinical Neurophysiology*.

[34] Burke D., Hallett M., Fuhr P., Pierrot-Deseilligny E. (1999). H reflexes from the tibial and median nerves. The International Federation of Clinical Neurophysiology. *Electroencephalography and Clinical Neurophysiology Supplement*.

[35] Wiley M. E., Damiano D. L. (1998). Lower-Extremity strength profiles in spastic cerebral palsy. *Developmental Medicine & Child Neurology*.

[36] Stackhouse S. K., Binder-Macleod S. A., Lee S. C. K. (2005). Voluntary muscle activation, contractile properties, and fatigability in children with and without cerebral palsy. *Muscle & Nerve*.

[37] Tedroff K., Knutson L. M., Soderberg G. L. (2008). Co-activity during maximum voluntary contraction: A study of four lower-extremity muscles in children with and without cerebral palsy. *Developmental Medicine & Child Neurology*.

[38] Unnithan V. B., Dowling J. J., Frost G., Bar-Or O. (1996). Role of cocontraction in the O2 cost of walking in children with cerebral palsy. *Medicine & Science in Sports & Exercise*.

[39] Gracies J.-M. (2005). Pathophysiology of spastic paresis. I: paresis and soft tissue changes. *Muscle & Nerve*.

[40] Barber L., Barrett R., Lichtwark G. (2011). Passive muscle mechanical properties of the medial gastrocnemius in young adults with spastic cerebral palsy. *Journal of Biomechanics*.

[41] Barber L., Hastings-Ison T., Baker R., Barrett R., Lichtwark G. (2011). Medial gastrocnemius muscle volume and fascicle length in children aged 2 to 5years with cerebral palsy. *Developmental Medicine & Child Neurology*.

[42] Barber L. A., Read F., Lovatt Stern J., Lichtwark G., Boyd R. N. (2016). Medial gastrocnemius muscle volume in ambulant children with unilateral and bilateral cerebral palsy aged 2 to 9 years. *Developmental Medicine & Child Neurology*.

[43] Dayanidhi S., Lieber R. L. (2014). Skeletal muscle satellite cells: Mediators of muscle growth during development and implications for developmental disorders. *Muscle & Nerve*.

[44] Truscelli D., Lespargot A., Tardieu G. (1979). Variation in the long term results of elongation of the tendo Achillis in children with cerebral palsy. *The Journal of Bone & Joint Surgery (British Volume)*.

[45] Swatland H. J. (1980). Volumetric growth of muscle fibers in ducks. *Growth*.

[46] Swatland H. J. (1980). Analysis of growth in a complex muscle (m. supracoracoideus, Anas platyrhynchos). *Growth*.

[47] Heslinga J. W., Huijing P. A. (1993). Muscle length-force characteristics in relation to muscle architecture: a bilateral study of gastrocnemius medialis muscles of unilaterally immobilized rats. *European Journal of Applied Physiology*.

[48] Heslinga J. W., te Kronnie G., Huijing P. A. (1995). Growth and immobilization effects on sarcomeres: a comparison between gastrocnemius and soleus muscles of the adult rat. *European Journal of Applied Physiology*.

[49] Zuurbier C. J., Heslinga J. W., Lee-de Groot M. B. E., Van der Laarse W. J. (1995). Mean sarcomere length-force relationship of rat muscle fibre bundles. *Journal of Biomechanics*.

[50] Boulay C., Jacquemier M., Castanier E. (2015). Planovalgus foot deformity in cerebral palsy corrected by botulinum toxin injection in the peroneus longus: Clinical and radiological evaluations in young children. *Annals of Physical and Rehabilitation Medicine*.

